# The *Salmonella* Specific, σ^E^-Regulated, STM1250 and AgsA, Function With the sHsps IbpA and IbpB, to Counter Oxidative Stress and Survive Macrophage Killing

**DOI:** 10.3389/fcimb.2019.00263

**Published:** 2019-07-23

**Authors:** Claire L. Hews, Emily J. Pritchard, Gary Rowley

**Affiliations:** School of Biological Sciences, University of East Anglia, Norwich, United Kingdom

**Keywords:** *Salmonella*, envelope stress, oxidative stress, *rpoE*, sHsp

## Abstract

The host presents an array of environments which induce bacterial stress including changes in pH, antimicrobial compounds and reactive oxygen species. The bacterial envelope sits at the interface between the intracellular and extracellular environment and its maintenance is essential for *Salmonella* cell viability under a range of conditions, including during infection. In this study, we aimed to understand the contribution of the σ^H^- and σ^E^-regulated small heat shock proteins IbpA, IbpB, and AgsA and the putative σ^E^-regulated stress response protein STM1250 to the *Salmonella* envelope stress response. Due to shared sequence identity, regulatory overlap, and the specificity of STM1250 and AgsA to *Salmonella* sp., we hypothesized that functional overlap exists between these four stress response proteins, which might afford a selective advantage during *Salmonella* exposure to stress. We present here new roles for three small heat shock proteins and a putative stress response protein in *Salmonella* that are not limited to heat shock. We have shown that, compared to WT, a quadruple mutant is significantly more sensitive to hydrogen peroxide, has a lower minimum bactericidal concentration to the cationic antimicrobial peptide polymyxin B, and is attenuated in macrophages.

## Introduction

*Salmonella* sp. are major causes of morbidity and mortality worldwide. In 2010, it was estimated that non-typhoidal *Salmonella* (NTS) was responsible for 93.9 million cases of disease and 155,000 deaths per year (Majowicz et al., 2010). Emphasizing the global significance of *Salmonella* infection, the World Health Organization priority pathogens list (Tacconelli et al., 2018) details fluoroquinolone resistant *Salmonella* to be of high priority for research. As a result, a better understanding of the mechanisms of *Salmonella* infection and survival in the host will direct research into new therapeutics for this prevalent pathogen.

During the *Salmonella* life cycle a range of precarious environments are encountered, both within the host and the environment, that have the potential to be bacteriostatic and/or bactericidal in nature. Such conditions need to be detected and transduced to allow appropriate transcriptional responses to be elicited that counter the stress. During mammalian infection, *Salmonella* encounters highly stressful environments; macrophages are sites of reactive oxygen species (ROS) and nitric oxide (NO), and intestinal antimicrobial peptides can disrupt the outer membrane (OM) causing serious cellular damage. The cell envelope, formed of the OM, periplasmic space, peptidoglycan layer, and inner membrane, is a crucial barrier between the bacteria and its extracellular environment. Thus, maintenance of this barrier is essential to survival and significantly contributes to the success of *Salmonella* as an intracellular pathogen.

The extracytoplasmic sigma factor σ^E^ (RpoE) (reviewed in Rowley et al., 2006), is an important gatekeeper for maintenance of the cell envelope, detecting stresses and subsequent perturbations to OM and periplasmic proteostasis. In the intracellular pathogen *Salmonella, rpoE* is upregulated within macrophages (Eriksson et al., 2003) and regulates genes required for resistance to oxidative stress and heat (Testerman et al., 2002). Moreover, it has been shown previously that σ^E^ is extremely important for *Salmonella* during intracellular survival in macrophages and in murine infection (Humphreys et al., 1999). Well-characterized σ^E^-regulated genes make significant contributions to envelope maintenance and the infection process (Humphreys et al., 2003; Lewis et al., 2009; Rowley et al., 2011). Additionally, in the serovar responsible for typhoid fever, *S*. Typhi, *rpoE* is important for intracellular invasion and survival (Zhang et al., 2016). Clearly, the σ^E^ regulon is of great importance to multiple stages of *Salmonella* host colonization across different serovars. That given, there is much we do not understand about the contribution of a number of σ^E^-regulated genes to the envelope stress response (ESR) and infection.

Identification of the σ^E^ regulon in *Salmonella* highlighted a number of genes of unknown function (Skovierova et al., 2006) including *ibpA, ibpB, agsA*, and the putative cytoplasmic protein *STM1250*. In addition to σ^E^ regulation, *ibpA, ibpB*, and *agsA* are also regulated by the sigma factor σ^H^. Although primarily induced by heat shock, members of the σ^H^ regulon have been linked to bacterial pathogenesis (Roncarati and Scarlato, 2017).

IbpA and IbpB (from herein referred to as IbpAB) are well-conserved across species of Gram-negative bacteria. These proteins share 50% amino acid sequence identity and were first observed in *E. coli* to be highly expressed and associated with inclusion bodies (inclusion body protein) during expression of heterologous proteins (Allen et al., 1992). As members of the small heat shock protein (sHsp) family, IbpA and IbpB are 15 and 16 kDa respectively and contain a C-terminal α-crystallin domain, a characteristic feature of sHsps (Nakamoto and Vígh, 2007). Furthermore, the *ibpA* 5′-untranslated region (UTR) encodes a ROSE (repression of heat shock gene expression)-like RNA thermometer enabling its temperature controlled expression (Waldminghaus et al., 2009). IbpAB have been shown to associate with endogenous proteins in *E. coli* following heat shock (Laskowska et al., 1996); however, limited phenotypic or functional studies exist for these proteins in *Salmonella* and to date no contribution to infection has been identified.

Conversely, STM1250 and AgsA are unique to *Salmonella* spp. (Skovierova et al., 2006). AgsA (aggregation-supressing protein A) is a 17 kDa sHsp with 32% amino acid sequence identity to IbpA and 31% to IbpB (Tomoyasu et al., 2003). Like *ibpA*, the *agsA* 5′-UTR contains an RNA thermometer for temperature control of expression (Waldminghaus et al., 2007). The 10 kDa protein STM1250 is not a member of the sHsp family, lacking the α-crystallin domain. However, *STM1250* has been shown to form an operon with *agsA* and the two genes are separated by only 151 bp (Skovierova et al., 2006).

In this study we aimed to understand the contribution of the stress-induced small heat shock proteins IbpA, IbpB, AgsA, and the *Salmonella* specific putative chaperone STM1250, to the ESR. Despite their description as sHsps we hypothesize that IbpAB and AgsA, in cooperation with STM1250, are involved in tolerance to multiple stresses. Interestingly, *ibpAB* and *STM1250* are highly expressed during intracellular infection and all genes are expressed under osmotic stress (Kröger et al., 2013; Canals et al., 2019). Additional published gene expression and TraDIS libraries provide interesting insights into the potential roles of these genes. Chaudhuri et al. (2013) identified that a *STM1251* TraDIS mutant is attenuated in cattle, but not chickens, swine or mice. Together, these data support that these genes may function beyond tolerance to heat shock.

Based on sequence identity, shared regulation by σ^E^ and σ^H^, and genomic location, we predict functional overlap exists between these proteins, and the putative stress responsive protein STM1250, that in *Salmonella*, is not limited to surviving heat shock. To investigate this, we have subjected deletion mutants to conditions known to perturb envelope homeostasis and present new roles for these stress responsive proteins. We have demonstrated that an Δ*ibpAB*Δ*STM1250*Δ*agsA* quadruple mutant is attenuated in macrophages and is more sensitive to hydrogen peroxide (H_2_O_2_) -induced oxidative stress and the cationic antimicrobial peptide polymyxin B, compared to WT.

## Materials and Methods

### Bacterial Strains and Growth Conditions

Bacterial strains used in this study are detailed in Table 1. Bacteria were maintained on LB agar and overnight cultures were grown in LB broth at 37°C with aeration, supplemented with 50 μg/mL kanamycin, 30 μg/mL chloramphenicol, or 100 μg/mL ampicillin where required. Bacterial growth curves were performed in 24 well plates in a SpectraMax M5 microplate reader with culture volumes of 1 mL. An overnight culture was diluted to OD_600_ 0.1 in LB. Where stated, 6 mM hydrogen peroxide (H_2_O_2_, Sigma), 1,000–4,000 U/mL bovine catalase (Sigma), 3 mM CuCl_2_, 0.5 μM potassium tellurite (K_2_TeO_3_), 30 mM paraquat (methyl viologen, Sigma), or 1 mM indole was added to each well. The plate was incubated at 37°C and OD_600_ reading taken every hour, with 3 s of agitation before each reading.

**Table 1:** Bacterial strains and plasmids used in this study.

**Strain**	**Description**	**Source**
**STRAINS**		
SL1344	*Salmonella enterica* serovar Typhimurium 4/74, *hisG, rpsL*	Hoiseth and Stocker, 1981
GVB2551	SL1344 Δ*fkpA*Δ*surA*Δ*ppiAD*	Gift from Professor Mark Roberts, University of Glasgow
Δ*ibpAB*	SL1344 Δ*ibpA-B* :: Kan	This study
Δ*STM1250*Δ*agsA*	SL1344 Δ*STM1250*Δ*agsA* :: Cm	This study
Δ*ibpAB*Δ*agsA*	SL1344 Δ*ibpA-B* :: Kan Δ*agsA* :: Cm	This study
Δ*ibpAB*	SL1344 Δ*ibpA-B* :: Kan Δ*STM1250*Δ*agsA* :: Cm	This study
Δ*STM1250*Δ*agsA*		
Δ*rpoE*	SL1344 Δ*rpoE* :: Kan	Humphreys et al., 1999
**PLASMIDS**		
pKD46	λ-Red helper plasmid carrying γ, β, and *exo* genes under control of P_araB_ promoter. Temperature sensitive replication. AmpR	Datsenko and Wanner, 2000
pKD3	pANT-Sγ derivative, FRT-flanked CmR	Datsenko and Wanner, 2000
pKD4	pANT-Sγ derivative, FRT-flanked KanR	Datsenko and Wanner, 2000

### Construction of Deletion Mutants

Mutants were generated by lambda Red recombination (Datsenko and Wanner, 2000). Chloramphenicol or kanamycin antibiotic resistance cassettes were amplified by PCR from the plasmids pKD3 or pKD4, respectively, using Biomix Red (Bioline) and mutant primers in Supplementary Table S1. The PCR product was transformed into SL1344 harboring the pKD46 plasmid. Transformants were screened by colony PCR using verification primers (Supplementary Table S1). Successful mutants were transduced into a clean WT background by P22 transduction. Triple and quadruple mutants were generated by transducing a Δ*agsA* or Δ*STM1250*Δ*agsA* mutation into the SL1344 Δ*ibpAB* background.

### Sequence Alignments

Amino acid sequences were retrieved from NCBI. Sequences were aligned using M-Coffee online alignment tool (Notredame et al., 2000) and shaded figure of multiple-alignments generated using the ExPASy BoxShade online tool (https://embnet.vital-it.ch/software/BOX_form.html).

### Temperature Shock Assays

Bacteria were cultured in LB at 37°C for 3 h to mid-log phase. Cultures were then incubated at 10 or 50°C. At each time point, a 1 mL sample was taken, serially diluted in PBS and spotted onto LB agar plates in 10 μL spots. Plates were incubated O/N at 37°C and percentage survival for each time point calculated by comparing to the 0-h non-shocked control.

### Determination of Minimum Bactericidal Concentration

Minimum bactericidal concentration (MBC) assays were performed in 96-well plates according to the Clinical and Laboratory Standards Institute guidelines. Briefly, an O/N culture was diluted to OD_600_ 0.08 in LB and 100 μL added to each well. Polymyxin B was added to a final concentration of either 16 or 12 μg/mL and 1:2 serial dilutions performed across the plate. Bacteria were incubated at 37°C O/N, for at least 18 h before reading OD_600_.

### Disc Diffusion and Vancomycin Sensitivity Assays

For disc diffusion assays, an O/N culture was diluted 1:100 in LB and incubated at 37°C with aeration for 1 h. A 0.75% (w/v) agarose top agar was prepared in LB, and 4 mL top agar inoculated with 100 μL culture. Sterile whatman discs were impregnated with 10 μL 10% (w/v) SDS, 1% (v/v) Triton X-100 or 3% (v/v) H_2_O_2_.

For vancomycin sensitivity assays, O/N cultures were standardized to OD_600_ 1.0, diluted to 1 × 10^−7^ CFU/mL and 10 μL spots of each dilution plated onto LB agar containing 65 μg/mL vancomycin hydrochloride (Alfa Aesar, Thermo Fisher Scientific).

### Gentamicin Protection Assay

RAW264.7 murine macrophages were maintained in Dulbecco's Modified Eagle's Medium (Invitrogen) supplemented with 10% fetal bovine serum (FBS) (Sigma) and 2 mM L-glutamine (Sigma). Cells were grown at 37°C in a 5% CO_2_ (v/v) atmosphere.

RAW264.7 were seeded into 24-well plates at a density of 10^6^ cells/mL and incubated for 3 h before addition of IFN-γ (1,000 U) for activation. Cells were grown for a further 21 h before infection. Bacteria were cultured on LB agar plates and macrophages infected with a bacterial dose at a multiplicity of infection of 10 to 1 cells. After 1 h of infection, cells were treated with 100 μg/mL gentamicin (Invitrogen) for 1 h. Samples for 2 h time point were washed twice in PBS, lysed with 1 mL of 1% (v/v) Triton X-100 and 0.1% (v/v) SDS in PBS and plated on LB agar for CFU/mL counts. Samples for 24 h time point were incubated in 10 μg/mL gentamicin for a further 22 h then lysed as previous. For inhibition of NADPH oxidase, 250 μM apocynin (acetovanillone, 4-hydroxy-3-methoxyacetophenon, Sigma) was added with the bacterial dose, with each gentamicin treatment and after 8 h for the 24 h infection samples.

### Statistical Analysis

Data were analyzed using GraphPad Prism version 8 software and statistical analysis performed by one-way ANOVA or student *t*-test, as stated in the figure legends.

## Results

### STM1250 and AgsA Have Divergently Evolved Across *Salmonella* Serovars

The sHsps *ibpA* and *ibpB* are highly conserved across all enteric Gram-negative bacteria; however, *STM1250* and *agsA* are unique to *Salmonella* (Skovierova et al., 2006). The *Salmonella* genus can be divided into two distinct species, *S. bongori* and *S. enterica*. While *S. bongori* rarely causes infection in humans and is a commensal of cold-blooded animals (Fookes et al., 2011), serovars of *S. enterica* subspecies *enterica* are responsible for infections in a broad range of mammalian hosts. These hosts include humans, chickens, swine and cattle. We sought to determine whether these *Salmonella* specific genes are conserved across different *Salmonella* serovars. Amino acid sequences obtained from the NCBI database were aligned using M-Coffee (Notredame et al., 2000) and Expasy Boxshade (https://embnet.vital-it.ch/software/BOX_form.html) online tools. Alignments are presented in Supplementary Figure S1.

AgsA is highly conserved and shown to be part of the core genome across all serovars tested in this study. Conversely, alignment of the amino acid sequence of *S*. Typhimurium STM1250 showed different levels of homology across the serovars tested. Between 99 and 100% identity was observed between *S*. Typhimurium, the invasive NTS serovar D23580 and the non-invasive serovar *S*. Enteritidis and 92% identity with *S*. Typhi. However, the *S*. Typhimurium STM1250 sequence was not found to be conserved in *S*. Paratyphi, *S*. Choleraesuis, *S*. Newport, or *S*. Dublin. As such, we cannot observe any patterns of identity that enable *STM1250* to be associated with a specific invasive/non-invasive disease type, although it appears that *STM1250* is more highly conserved in serovars which cause disease in humans rather than other mammals. Interestingly, *S. bongori STM1250* is annotated as a pseudogene suggesting that functional *STM1250* arose after divergence of the *S. enterica* species. These data point toward a role for *STM1250* in *S. enterica* mammalian infection.

### *Salmonella* Survival at 50 and 10°C Requires IbpAB and AgsA but Not STM1250

The IbpAB sHsps have been shown to bind to aggregated proteins in *E. coli* following heat shock (Laskowska et al., 1996); however, they are not essential for survival at 50°C (Thomas and Baneyx, 1998). We aimed to determine whether this is also true for *Salmonella ibpAB*, and furthermore, determine whether survival of an *STM1250agsA* double mutant is affected at high temperatures. STM1250 is not currently described as a sHsp; however, Hsu-Ming et al. (2012) identified that *STM1250* is upregulated during recovery from heat shock at 55°C.

**Figure 1:**
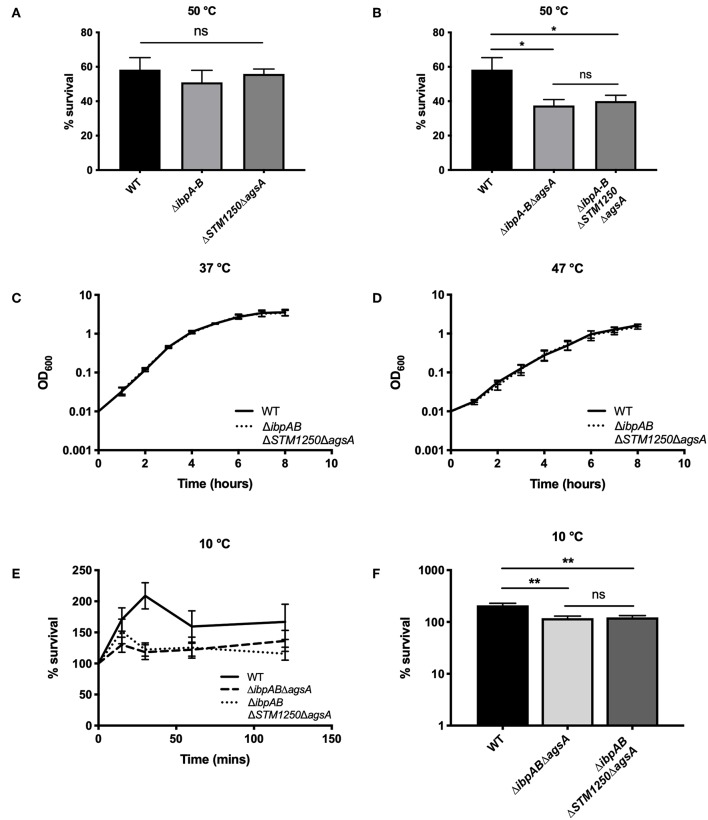
*ibpAB* and *agsA* contribute to *S*. Typhimurium survival following sudden changes in temperature. **(A)** Percentage survival following a 4 h incubation at 50°C. Bacteria were grown for 3 h at 37°C before incubation at 50°C. Survival of the double mutants are equal to WT. **(B)** Percentage survival following a 4 h incubation at 50°C. Bacteria were grown for 3 h at 37°C before incubation at 50°C. Survival of the triple and quadruple mutant were significantly reduced compared to WT. **(C)** Growth of WT and the quadruple mutant are equivalent at 37°C and **(D)** 47°C. **(E)** Percentage survival over a 2 h incubation at 10°C. Bacteria were grown for 2 h at 37°C before incubation at 10°C. **(F)** Survival of the triple and quadruple mutant is significantly reduced after 30 min at 10°C. Data are the means of three separate experiments performed in duplicate. Error bars represent SEM. Data analyzed by one-way ANOVA with Tukey's multiple comparisons test, ^*^p < 0.05 and ^**^p < 0.005.

Bacteria were grown to mid-log phase at 37°C and then transferred to a water bath at 50°C. In agreement with previous studies in *E. coli* (Thomas and Baneyx, 1998), the Δ*ibpAB* double mutant was unaffected at 50°C. Furthermore, survival of the Δ*STM1250*Δ*agsA* double mutant was also equivalent to WT (Figure 1A). As a result, we constructed triple (Δ*ibpAB*Δ*agsA*) and quadruple (Δ*ibpAB*Δ*STM1250*Δ*agsA*) deletion mutants to test our proposed hypothesis of functional redundancy. Survival of the triple deletion mutant in *Salmonella* was compromised after heat shock (Tomoyasu et al., 2003). Our data agreed with this finding; survival of the triple mutant was significantly reduced compared to WT at 50°C (Figure 1B). Survival of the quadruple mutant was not significantly different to the triple mutant (Figure 1B), suggesting there is no additive contribution of *STM1250* to surviving heat shock at this temperature.

The ability of the quadruple deletion mutant to grow at high temperature, rather than survive shock, was also tested. We observed that at 37 and 47°C, the mutant grew equally to WT (Figures 1C,D) suggesting that the reduced survival phenotype following heat shock, resulting from loss of *ibpAB* and *agsA*, is limited to perturbations induced by sudden heat shock or at least sudden changes in temperature. Considering this, we also investigated the ability of the triple and quadruple deletion mutants to survive cold shock at 10°C. Cold shock is one of the major inducers of *rpoE* (Miticka et al., 2003) and incubation at low temperatures is known to disrupt bacterial membrane fluidity (Barria et al., 2013). Over the course of 2 h, growth of all strains was observed (Figure 1E). However, there was a significant increase, particularly after 30 min, in WT growth compared to the triple and quadruple mutant (Figure 1F), but again no significant difference between the triple and quadruple mutant, indicating no role for STM1250 in responding to temperature-based damage.

### Deletion of the σ^E^-Regulated *ibpAB, STM1250* and *agsA* Does Not Impact Overall OM Integrity in *Salmonella*

**Figure 2:**
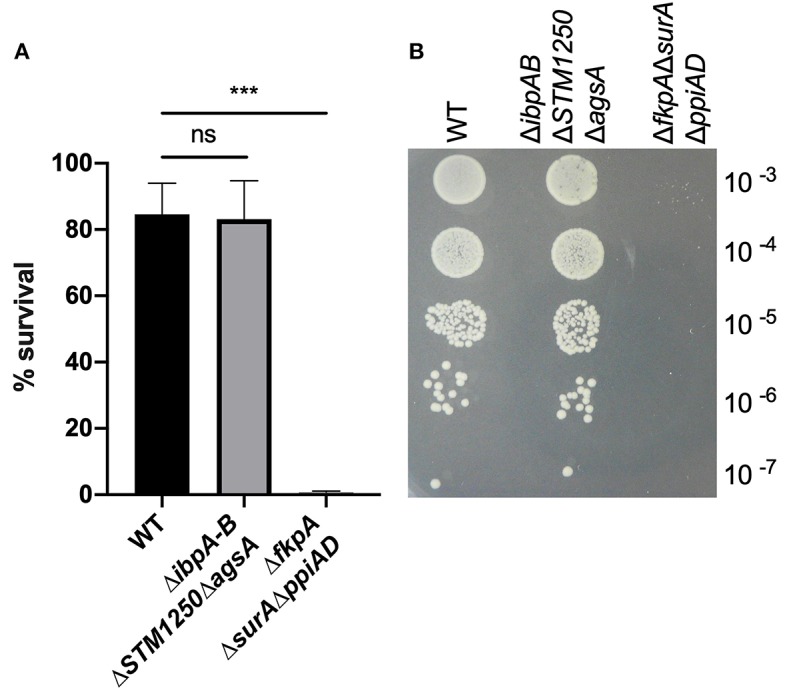
The quadruple deletion mutant is not sensitive to 65 μg/mL vancomycin. O/N cultures were serially diluted in PBS and 10 μL spotted onto LB agar either with or without 65 μg/mL vancomycin. **(A)** Colonies grown on vancomycin expressed as a percentage of colonies on LB agar. **(B)** Representative image of growth on vancomycin. Data are the means of three separate experiments performed in duplicate. Error bars show SEM. Data analyzed by one-way ANOVA with Tukey's multiple comparisons test, ^***^p < 0.0005.

The proteins in this study are predicted to be cytoplasmic, although *ibpA* has been detected in the OM and S-fraction of *E. coli* following heat shock (Kuczynska-Wisnik et al., 2002). Moreover, due to their regulation by σ^E^, we hypothesized that *ibpA, ibpB, STM1250*, and *agsA* may be involved in maintaining OM integrity. We tested this by measuring the survival of WT and Δ*ibpAB*Δ*STM1250*Δ*agsA* after exposure to 65 μg/mL vancomycin, 1% Triton X-100 and 10 % SDS, compounds known to be excluded by an intact OM. Both WT and the quadruple mutant were resistant to Triton X-100 and SDS in disc diffusion assays (data not shown). Vancomycin is unable to traverse the Gram-negative bacterial membrane; however, the deletion of σ^E^-regulated chaperones (Δ*fkpA*Δ*ppiAD*Δ*surA*) has been shown to increase sensitivity in *E. coli* via loss of maintenance of the cell envelope (Justice et al., 2005). No significant difference was observed in the ability of Δ*ibpAB*Δ*STM1250*Δ*agsA* to grow on LB agar containing vancomycin; however, survival of Δ*fkpA*Δ*ppiAD*Δ*surA* was significantly reduced (Figure 2). These data suggest that no overall alteration to the permeability or integrity of the OM is caused by the loss of the IbpAB, STM1250, and AgsA stress responsive proteins.

### Lag Phase of the Δ*ibpAB*Δ*STM1250*Δ*agsA* Mutant Is Extended by H_2_O_2_ Oxidative Stress

Heat shock proteins are known to overlap in function with hydrogen peroxide (H_2_O_2_) induced proteins (Morgan et al., 1986) and the σ^E^ regulon is important for *Salmonella* resistance to oxidative stress (Testerman et al., 2002). Both *ibpA* and *ibpB* are strongly induced by H_2_O_2_ in *E. coli* (Zheng et al., 2001). However, to date, there is no specific link between sHsps and tolerance to oxidative stress in *Salmonella*. We aimed to determine whether the proteins of interest in this study are involved in resistance to oxidative stress using H_2_O_2_.

**Figure 3:**
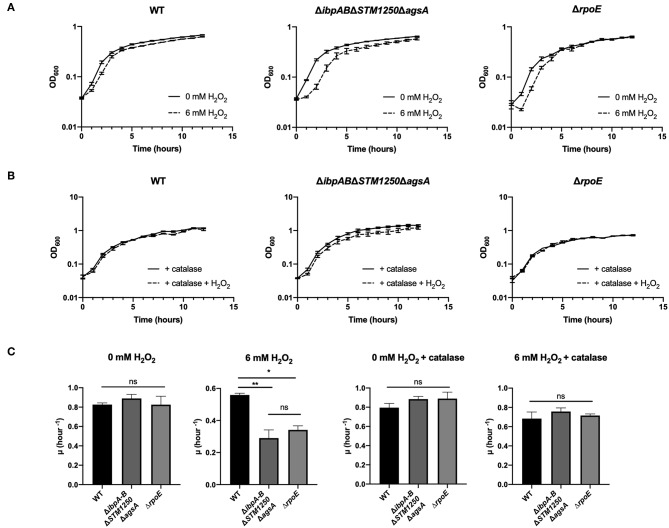
The quadruple deletion mutant is more sensitive to 6 mM H_2_O_2_ compared to WT. Bacteria were grown in a plate reader for 12 h at 37°C. **(A)** Bacteria grown either with or without 6 mM H_2_O_2_. OD_600_ readings taken hourly. **(B)** Bacteria grown in the presence of 1,000–4,000 U/mL bovine catalase and either with or without 6 mM H_2_O_2_. OD_600_ readings taken hourly. **(C)** Initial growth rate constants for bacterial growth in the presence/absence of 6 mM H_2_O_2_ and/or 1,000–4,000 U/mL bovine catalase. Data are the means of three separate experiments performed in duplicate. Error bars show SEM. Data analyzed by one-way ANOVA with Tukey's multiple comparisons test, ns *p* > 0.05, ^*^p < 0.05 and ^**^p < 0.005 vs. WT.

Bacteria were grown in a plate reader in the presence of 6 mM H_2_O_2_ and the OD_600_ read hourly. The quadruple mutant showed the greatest sensitivity to H_2_O_2_ compared to WT (Figure 3A and Supplementary Figure S2) with an extended lag phase, indicative of H_2_O_2_-induced stress during bacterial growth (Watson and Schubert, 1969). This phenotype was also observed for the Δ*rpoE* control strain and the growth rate constant of the quadruple mutant and Δ*rpoE* control was significantly reduced in the first 2 h compared to WT (Figure 3C). Growth of all strains was restored to non-treated levels by the addition of the hydrogen peroxide scavenger, bovine catalase (Figures 3B,C).

**Figure 4:**
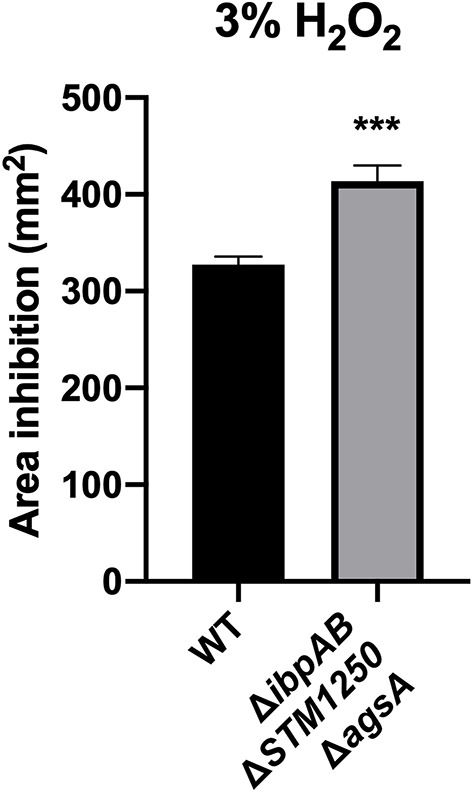
The quadruple mutant is more sensitive to 3% H_2_O_2_. Bacteria were cultured in top agar on LB and sterile discs impregnated with 3% H_2_O_2_. Data are the means of three separate experiments performed in triplicate. Error bars show SEM. Data analyzed by Student's *t*-test, ^***^p < 0.0005.

Sensitivity to H_2_O_2_ was also tested by disc diffusion assay. In agreement with growth curve findings, the quadruple mutant was more sensitive to 3% H_2_O_2_ compared to WT with a significant difference in the area of inhibition (Figure 4).

**Figure 5:**
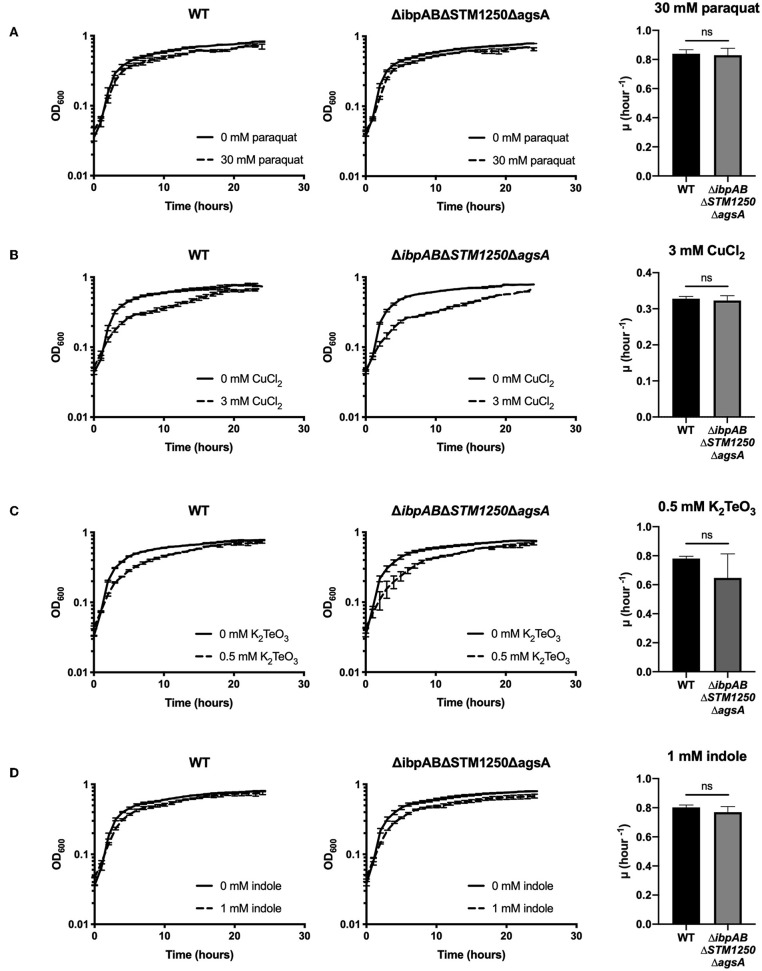
Deletion of ibpAB, STM1250, and agsA has no effect on overall growth or initial growth rate (μ hour^−1^) in the presence of 30 mM paraquat, 3 mM CuCl2, 0.5 μM K2TeO3, or 1 mM indole. Bacteria were grown in a plate reader at 37°C. Graphs show growth over 24 h or initial growth rate for WT and the quadruple mutant in the presence of **(A)** 30 mM paraquat (methyl viologen) **(B)** 3 mM CuCl2 **(C)** 0.5 μM K2TeO3 or **(D)** 1 mM indole. Data are the means of three separate experiments performed in duplicate. Error bars represent SEM. Data analyzed by one- way ANOVA with Tukey's multiple comparisons test, ns *p* > 0.05.

Previously, *ibpAB* have been linked to oxidative stress resistance in *E. coli* (Kitagawa et al., 2000, 2002) and an *E. coli ibpAB* mutant is more sensitive to CuCl_2_-induced oxidative stress (Matuszewska et al., 2008). Moreover, the *ibpA* promoter is more active in the presence of potassium tellurite (K_2_TeO_3_), a further inducer of bacterial oxidative stress (Pérez et al., 2007). Since these studies were performed on *E. coli*, we sought to determine whether the *Salmonella* Δ*ibpAB* mutant and quadruple deletion strain in this study were sensitive to CuCl_2_ or K_2_TeO_3_. In addition, in order to further determine whether the increased sensitivity to oxidative stress of the Δ*ibpAB*Δ*STM1250*Δ*agsA* mutant is limited to H_2_O_2_, bacteria were also challenged with the oxidizing agent methyl viologen (paraquat). *Salmonella* was grown in the presence of 30 mM paraquat (Figure 5A), 3 mM CuCl_2_ (Figure 5B), or 0.5 μM K_2_TeO_3_ (Figure 5C) for 24 h. We observed no differences in sensitivity or initial growth rate constant (μ h^−1^) between WT or the quadruple mutant for these alternative bio-oxidants.

Additionally, in a previous study, *agsA* was found to be upregulated in the presence of 1 mM indole (Nikaido et al., 2012); however, no specific contribution of *agsA* to tolerance of increased levels of indole has been shown. Indole is produced by bacteria from tryptophan by tryptophanase (TnaA). *Salmonella* lacks the TnaA enzyme and is, therefore, unable to produce indole. Interestingly, Garbe et al. (2000) showed that exposure to indole induced expression of antioxidant proteins in *E. coli*. The proposed mechanism described that the lipophile indole dissolves within membrane lipids, affecting membrane integrity and enabling quinones to interact with oxygen, subsequently leading to the generation of superoxide (Garbe et al., 2000). In the current study, exposure of WT and mutant *Salmonella* to 1 mM indole did not significantly affect the growth rate constant or overall growth over 24 h (Figure 5D).

### The Quadruple Mutant Is More Sensitive to Polymyxin B With a Lower MBC Range Than WT *S*. Typhimurium

Polymyxin B is a cationic antimicrobial peptide and binds to the negative charge of lipopolysaccharide (LPS) on the bacterial cell surface, causing disruption of the outer membrane. During host infection, cationic antimicrobial peptides (cAMPs) are released by intestinal epithelial cells (IECs) as part of the host immune defense (reviewed in Muniz et al., 2012). As a result, resistance to cAMPs is highly important to enteric bacteria.

**Figure 6:**
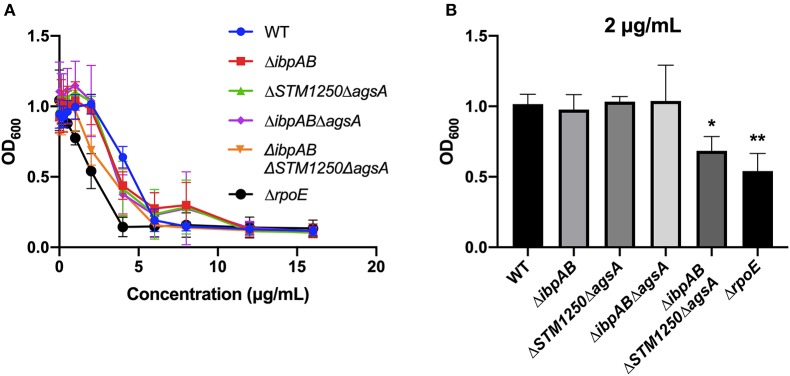
Deletion of *ibpAB, STM1250*, and *agsA* increases sensitivity of *Salmonella* to polymyxin B. **(A)** MBC assays were performed in 96-well plates containing different concentrations of polymyxin B. **(B)** A 2 μg/mL concentration of polymyxin B significantly limited the overall growth of Δ*ibpAB*Δ*STM1250*Δ*agsA* and an Δ*rpoE* control. Data are the means of three separate experiments performed in quadruplicate. Data analyzed by one-way ANOVA with Dunnett's multiple comparisons test, ^*^p < 0.05, ^**^p < 0.005 vs. WT.

Polymyxin B MBC assays were performed with WT and mutant strains. After an O/N incubation in a range of polymyxin B concentrations, the quadruple mutant and Δ*rpoE* control had a lower MBC range than WT (Figure 6A and Supplementary Table S2). In addition, overall growth of the quadruple mutant and Δ*rpoE* was limited significantly at 2 μg/mL compared to WT (Figure 6B) while no significant differences were observed between WT and the remaining mutants tested.

### IbpAB, STM1250, and AgsA Are Critically Important for *Salmonella* Intracellular Survival

Resistance to oxidative stress is an important contributor to the ability of *Salmonella* to cause infection. σ^E^-regulated genes are important for *Salmonella* intracellular survival (Humphreys et al., 1999). As indicated in Figure 1, increased expression of *ibpAB, STM1250*, and *agsA* has been previously observed during intracellular macrophage infection and further studies have indicated their upregulation during intracellular survival (Eriksson et al., 2003).

**Figure 7:**
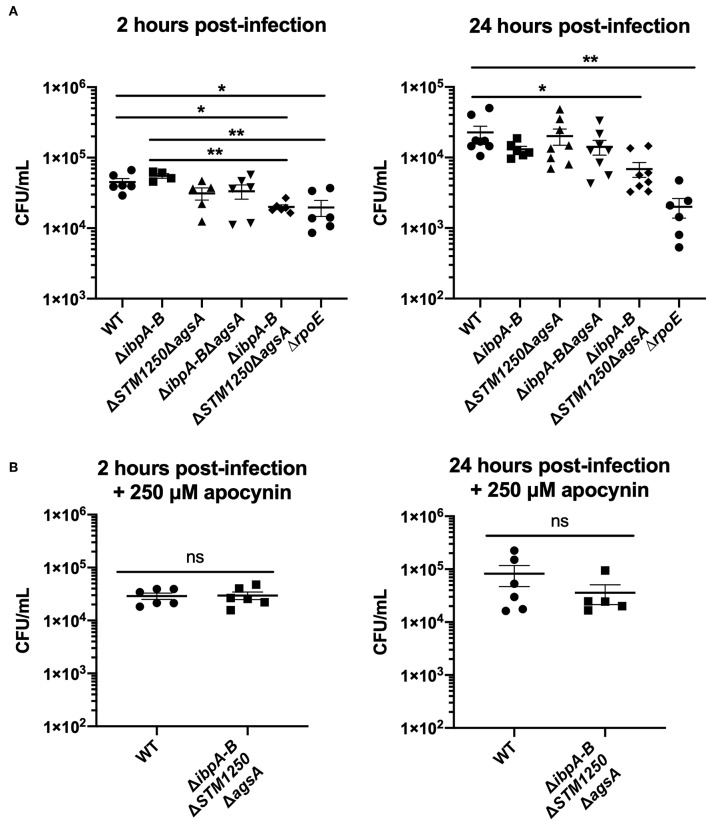
The quadruple deletion mutant is attenuated in IFN-γ activated RAW264.7 macrophages after 2 and 24 h of infection but can be rescued by inhibition of the macrophage NADPH oxidase. **(A)** Macrophages infected for 2 or 24 h, data represents intracellular CFU/mL at each time point. **(B)** IFN-γ activated macrophages in the presence of apocynin, infected for 2 and 24 h. Data are the means of three separate experiments performed in duplicate. Error bars show SEM. Data analyzed by **(A)** one-way ANOVA with Tukey's multiple comparisons test, ^*^p < 0.05, ^**^p < 0.005, **(B)** Student's *t*-test, ns *p* > 0.05.

In this study, IFN-γ activated RAW264.7 macrophages were infected with bacteria at an MOI of 10:1 and the number of intracellular CFU/mL determined at 2 and 24 h post-infection. Indeed, survival of the quadruple mutant was reduced compared to WT after 2 and 24 h of infection with significantly lower intracellular CFUs/mL (Figure 7A). Interestingly, after 2 h of infection, intracellular CFU/mL of Δ*ibpAB*Δ*STM1250*Δ*agsA* and Δ*rpoE* were also significantly reduced compared to Δ*ibpAB*, indicating a functional role for STM1250/AgsA in compensating for loss of IbpAB.

Macrophages are known to release ROS as a bactericidal mechanism and, therefore, our H_2_O_2_-sensitive quadruple mutant strain may be more susceptible to ROS killing within the macrophage. To investigate this, the macrophage NADPH oxidase was inhibited with 250 μM apocynin. Following a 2 and 24 h infection, the survival of the quadruple mutant was restored to WT levels (Figure 7B).

## Discussion

Detection of stresses and subsequent responses, to nullify and repair stress-induced damage, are critical to the ability of *S*. Typhimurium to cause infection. The envelope stress response regulator, σ^E^, required for *Salmonella* survival in macrophages and a mouse typhoid model (Humphreys et al., 1999), controls expression of a wide array of genes (Skovierova et al., 2006), which must, therefore, include systems which contribute to infection. However, many of the σ^E^-regulated genes of *Salmonella* are of unknown function. These genes may be of yet unrecognized importance to bacterial stress survival and pathogenesis, particularly where functional overlap exists, a common emerging theme in stress response biology. In this study, we aimed to investigate the roles of the σ^E^- and σ^H^-regulated IbpA, IbpB, and AgsA and σ^E^-regulated STM1250 in *S*. Typhimurium. Due to shared regulation and sequence identity, we hypothesized that functional overlap occurs between these proteins. We predicted that limited understanding of the roles of these proteins in *Salmonella* exists, because when single deletions are made no phenotypes are observed. To counter this, in this study, we have generated mutants incorporating deletions in up to four of the genes of interest to enable a better understanding of their roles during *Salmonella* stress survival and infection.

IbpA has been implicated with heat shock survival in other species, with a *Pseudomonas putida ibpA* mutant presenting a growth defect at 40°C (Krajewski et al., 2013). However, our *S*. Typhimurium Δ*ibpAB* mutant did not show reduced survival at 50°C, in agreement with previous studies in *E. coli* (Thomas and Baneyx, 1998). Additionally, it has been shown previously that a *Salmonella* Δ*agsA* mutant is unaffected by heat shock at 70°C (Tomoyasu et al., 2003) and in this study we found that an Δ*STM1250*Δ*agsA* double mutant survival is equal to WT and Δ*ibpAB* at 50°C. However, when a triple mutant, Δ*ibpAB*Δ*agsA* was subjected to heat shock, survival rate compared to WT was significantly reduced. In order to further investigate proposed functional redundancy, we also subjected a Δ*ibpAB*Δ*STM1250*Δ*agsA* quadruple mutant to heat shock and observed a significant decrease in survival compared to WT but no further reduction compared to the triple mutant. These data suggested that STM1250 does not function as a sHsp in *Salmonella*. This finding can largely be explained by the fact that STM1250 does not possess the characteristic α-crystallin domain seen in sHsps. We observed that the quadruple mutant grew equally as well as WT at elevated temperature and, therefore, proposed that reduced survival was only a result of sudden changes in temperature. Indeed, following cold shock at 10°C, the triple and quadruple mutant showed significantly reduced growth compared to WT. Cold shock has been shown to induce phospholipid phase separation and subsequently decrease membrane fluidity (Barria et al., 2013). Therefore, the proteins in this study may function to maintain integrity and fluidity during extreme temperature changes, although, the precise mechanism behind this in *Salmonella* remains unclear.

Due to previous studies linking sHsps to oxidative stress resistance and the σ^E^ regulon of importance for survival against oxidative stress, we proposed that functional overlap may occur in the presence of oxidizing agents. We observed a reduced rate of growth of our Δ*ibpAB*Δ*STM1250*Δ*agsA* mutant during the first 2 h in the presence of hydrogen peroxide. *ibpAB* have been linked to oxidative stress survival in a number of species including *E. coli* (Kitagawa et al., 2002) and *Yersinia pestis* (Pradel et al., 2014). Interestingly, van der Heijden et al. (2016) showed reduced survival of *Salmonella* Δ*ibpA* following a 2 h challenge with H_2_O_2_. We did not observe any differences between WT and *ibpAB* in this study; these conflicting findings may be due to the parent strain used. The previous study utilized an HpxF^−^ catalase and peroxidase negative background strain and this would certainly affect the response to H_2_O_2_ in WT. IbpA is proposed to bind to OmpC in *E. coli* (Butland et al., 2005). Moreover, an increase in H_2_O_2_ influx in an *S*. Typhimurium *ibpA* mutant has been shown and this was dependent on the OmpC porin (van der Heijden et al., 2016). In our study, deletion of *ibpA*, in conjunction with *ibpB, STM1250*, and *agsA*, may result in a higher influx of H_2_O_2_, which coupled to deletion of *ibpB, STM1250*, and *agsA* could explain the reduced growth rate of the quadruple mutant. Future work is required to elucidate the mechanism behind the contribution of STM1250 and AgsA to tolerance of H_2_O_2_ stress and it would be interesting to investigate *STM1250* expression in the triple mutant. Intracellular ROS and protein oxidation levels could also be measured for the different mutants to investigate H_2_O_2_ uptake and the downstream effects in the absence of the stress response proteins. Interestingly, exposure to alternative inducers of oxidative stress, CuCl_2_, K_2_TeO_3_, paraquat, and indole, did not affect the growth of the Δ*ibpAB*Δ*STM1250*Δ*agsA* mutant. These findings suggest that the sensitivity of the quadruple mutant is specifically limited to oxidative stress induced by H_2_O_2_ in the growth conditions tested in this study.

The mutants in this study were not sensitive to the OM-targeting compounds vancomycin, SDS or Triton X-100 detergents. The cAMP polymyxin B can also be used to investigate OM integrity as it binds to the cell surface LPS and subsequently disrupts the OM. σ^E^ and the two-component system PhoPQ regulate genes needed for resistance to cAMPs (Matamouros and Miller, 2015). IbpAB have been shown to associate with the OM (Laskowska et al., 1996) and a PhoP motif has been identified upstream of *STM1250* in *S*. Typhimurium (Monsieurs et al., 2005). We investigated whether the mutants generated in this study were susceptible to polymyxin B. MBC analyses identified that the quadruple mutant has a polymyxin B MBC in the range of 2–4 μg/mL while the MBC for the WT is between 1 and 2 μg/mL. Subsequently, overall growth of the quadruple mutant was significantly lower than WT in the presence of 2 μg/mL polymyxin B. Previous studies have shown that the LPS profile of an Δ*rpoE* mutant does not differ in comparison to the WT (Humphreys et al., 1999) and further work will be required to determine whether this is also the case for the Δ*ibpAB*Δ*STM1250*Δ*agsA* mutant in the current study. Interestingly, previous studies in *Acinetobacter baumannii* have identified that cAMPs such as polymyxin B can induce the production of intracellular hydroxyl radicals (OH^•^) (Sampson et al., 2012). Further work will be required to determine the levels of intracellular OH^•^ in the WT and mutant strains in this study, following cAMP treatment, to investigate whether the limited growth in the presence of polymyxin B is linked to the oxidative stress phenotypes we have reported.

Multiple studies have indicated a role for *ibpAB, STM1250*, and *agsA* during intracellular survival. Previously published expression data highlighted high expression of *ibpAB* and *STM1250* within macrophages (Canals et al., 2019) and *agsA* has also been observed to be upregulated over the course of a 21 h macrophage infection (Eriksson et al., 2003). Furthermore, overexpression of *ibpAB* in a non-pathogenic strain of *E. coli* was shown to increase survival of this strain within macrophages (Goeser et al., 2015). Macrophages are sites of oxidative and nitrosative stress. The NADPH-dependent oxidase Phox produces superoxide while the inducible nitric oxide synthase (iNOS) is responsible for the production of nitric oxide. In this study, survival of the Δ*ibpAB*Δ*STM1250*Δ*agsA* mutant was significantly reduced after 2 and 24 h of infection. Additionally, the intracellular CFU/mL of the quadruple mutant was also significantly lower than that of the Δ*ibpAB* mutant after 2 h. This is indicative or functional redundancy between these two stress response operons and is a possible reason for the lack of data on these genes via global mutagenesis studies. Together, with the slow growth in the presence of H_2_O_2_, we hypothesized that the reduction in survival could be, in part, due to macrophage induced oxidative stress. Apocynin was used to inhibit NADPH oxidase activity, and led to loss of the intracellular survival phenotype observed for the quadruple mutant in untreated cells. This chemical complementation indicates that IbpAB, STM1250, and AgsA function to protect *Salmonella* against macrophage induced oxidative stress. In addition to an involvement in protection against ROS, all genes of interest in this study have been previously shown to be upregulated by nitric oxide exposure (Richardson et al., 2011). Further work is required to determine whether our quadruple mutant is affected by exposure to nitrosative stress as well as oxidative stress.

The genomic location of *STM1250* and *agsA* may provide insight into their roles in *S*. Typhimurium. Upstream of *STM1250* is S*almonella* Pathogenicity Island 11 (SPI-11) (*STM1239, envF, msgA, envE, cspH, pagD, pagC, and pliC*). These proteins are highly important to virulence, with PagC required for serum resistance in *S*. Choleraesuis (Nishio et al., 2005) and PagD and MsgA important for intracellular survival (Gunn et al., 1995). Furthermore, genes within SPI-11 are regulated by σ^E^ and PhoPQ ESRs. Interestingly, *STM1250* is also regulated by Fis, a global regulator of virulence genes (Wang et al., 2013). The regulation of *STM1250* and *agsA*, as well as their proximity to SPI-11, points toward their role in infection. Interestingly, Canals et al. (2019) identified upregulation of *STM1250* in macrophages and, furthermore, observed a 4-fold increase in expression of *STM1250* in the invasive NTS (iNTS) serovar ST313. We suggested that STM1250 could not be associated with disease type based on our sequence homology analysis yet these data do suggest a role for STM1250 for iNTS serovars. This will require further investigation using a ST313 *STM1250* deletion strain.

In summary, this paper indicates new roles for sHsps, aside from resistance to heat shock stress. Overall, we demonstrate that these sHsps should instead be described as stress responsive proteins. We have demonstrated functional overlap between IbpA, IbpB, STM1250, and AgsA and for the first time have identified a role for these proteins during *Salmonella* intracellular survival. Ongoing work will include further study of STM1250, including biochemical characterization to better elucidate the mechanism by which these proteins function to tolerate oxidative stress and intracellular survival. Ultimately, an improved understanding of the role of stress response proteins during infection and survival of environmental stress, with particular emphasis on those with functional overlap, will identify whether these proteins are novel therapeutic targets.

## Data Availability

All datasets generated for this study are included in the manuscript and/or the Supplementary Files.

## Author Contributions

CH and GR designed the study. CH and EP performed the experimental work and CH analyzed the data. CH and GR wrote the manuscript. All authors read and reviewed the submitted manuscript.

### Conflict of Interest Statement

The authors declare that the research was conducted in the absence of any commercial or financial relationships that could be construed as a potential conflict of interest.
