# Supplementary material for: The Salmonella Specific, σE-Regulated, STM1250 and AgsA, Function With the sHsps IbpA and IbpB, to Counter Oxidative Stress and Survive Macrophage Killing
Source: Front Cell Infect Microbiol. 2019 Jul 23;9:263. doi: 10.3389/fcimb.2019.00263 (PMC6663981; doi:10.3389/fcimb.2019.00263)
Supplement: Supplementary file 1 [file Data_Sheet_1.docx]

**Table S1. Primer sequences used in this study for generation and verification of mutant strains.**

| **Primer name** | **Sequence (5’-3’)** |
| --- | --- |
| ibpAB_mutF | atgatggaaggtctgacattctcgctgatttcaggagtttgtgtaggctggagctgcttc |
| ibpAB_mutR | aaagccccgccatctctggcggggcaaggcaaggagctcacatatgaatatcctccttag |
| STM1250agsA_mutF | ataagccagttttttgtttcagcggtgactgaaacatattgtgtaggctggagctgcttc |
| STM1250agsA_mutR | gacaatgaaggcccgtctaaacgggcctccattaacgcgacatatgaatatcctccttag |
| agsA_mutF | gccttgatgttgaacttttgaatagtgattcaggaggttagtgtaggctggagctgcttc |
| agsA_mutR | gacaatgaaggcccgtctaaacgggcctccattaacgcgacatatgaatatcctccttag |
| ibpAB_verF | ttaacaatcagtgctgtccc |
| ibpAB_verR | atatcactcatcttttcttc |
| STM1250agsA_verF | aaattccagtcagttggcaa |
| STM1250agsA_verR | tttgtccactctcgacattc |

**A**

**B**

**Supplementary Figure S1. Amino acid alignment of (A) STM1250 and (B) AgsA across *Salmonella enterica* ssp. *enterica* serovars.** Alignments performed using M-coffee online alignment tool and figure generated using ExPasy Boxshade online tool.

**Supplementary Figure S2. Growth of double ∆*ibpAB*, ∆*STM1250*∆*agsA* and triple ∆*ibpAB*∆*agsA* deletion mutants are not significantly affected by 6 mM H_2_O_2_.** Bacteria were grown in a plate reader for 12 hours either with or without 6 mM H_2_O_2_ at 37 °C. **(A)** OD_600_ readings were taken hourly. **(B)** Initial growth rate constant from 0 h to 2 h in the absence or presence of 6 mM H_2_O_2_. Data are the means of three separate experiments performed in duplicate. Data analysed by one-way ANOVA with Tukey’s multiple comparisons test, ns p > 0.05.

**Supplementary Table S2. Strain minimal bactericidal concentration (MBC) ranges of polymyxin B in LB.**

| **Strain** | **MBC range (μg/mL)** |
| --- | --- |
| WT | 2 - 4 |
| ∆*ibpAB*∆*STM1250*∆*agsA* | 1 - 2 |
| ∆*rpoE* | 0.25 - 0.5 |
